# Bilateral cerebellar activation in unilaterally challenged essential tremor

**DOI:** 10.1016/j.nicl.2015.12.011

**Published:** 2015-12-28

**Authors:** Marja Broersma, Anna M.M. van der Stouwe, Arthur W.G. Buijink, Bauke M. de Jong, Paul F.C. Groot, Johannes D. Speelman, Marina A.J. Tijssen, Anne-Fleur van Rootselaar, Natasha M. Maurits

**Affiliations:** a Department of Neurology, University Medical Center Groningen, University of Groningen, PO Box 30001, 9700 RB Groningen, the Netherlands; b Neuroimaging Center, University Medical Center Groningen, University of Groningen, PO Box 30001, 9700 RB Groningen, the Netherlands; c Department of Neurology and Clinical Neurophysiology, Academic Medical Center, University of Amsterdam, PO Box 22660, 1100 DD Amsterdam, the Netherlands; d Brain Imaging Center, Academic Medical Center, University of Amsterdam, PO Box 22660, 1100 DD Amsterdam, the Netherlands; e Department of Radiology, Academic Medical Center, University of Amsterdam, PO Box 22660, 1100 DD Amsterdam, the Netherlands

**Keywords:** Essential tremor, FMRI, EMG, Cerebellum

## Abstract

**Background:**

Essential tremor (ET) is one of the most common hyperkinetic movement disorders. Previous research into the pathophysiology of ET suggested underlying cerebellar abnormalities.

**Objective:**

In this study, we added electromyography as an index of tremor intensity to functional Magnetic Resonance Imaging (EMG-fMRI) to study a group of ET patients selected according to strict criteria to achieve maximal homogeneity. With this approach we expected to improve upon the localization of the bilateral cerebellar abnormalities found in earlier fMRI studies.

**Methods:**

We included 21 propranolol sensitive patients, who were not using other tremor medication, with a definite diagnosis of ET defined by the Tremor Investigation Group. Simultaneous EMG-fMRI recordings were performed while patients were off tremor medication. Patients performed unilateral right hand and arm extension, inducing tremor, alternated with relaxation (rest). Twenty-one healthy, age- and sex-matched participants mimicked tremor during right arm extension. EMG power variability at the individual tremor frequency as a measure of tremor intensity variability was used as a regressor, mathematically independent of the block regressor, in the general linear model used for fMRI analysis, to find specific tremor-related activations.

**Results:**

Block-related activations were found in the classical upper-limb motor network, both for ET patients and healthy participants in motor, premotor and supplementary motor areas. In ET patients, we found tremor-related activations bilaterally in the cerebellum: in left lobules V, VI, VIIb and IX and in right lobules V, VI, VIIIa and b, and in the brainstem. In healthy controls we found simulated tremor-related activations in right cerebellar lobule V.

**Conclusions:**

Our results expand on previous findings of bilateral cerebellar involvement in ET. We have identified specific areas in the bilateral somatomotor regions of the cerebellum: lobules V, VI and VIII.

## Highlights

• We added EMG as an index of tremor intensity to fMRI to study essential tremor.
• Block- and tremor-related activations during a unilateral motor task were separated.
• Block-related activations were found in the classical motor network.
• Tremor-related activations were found in bilateral cerebellar lobules V, VI and VIII.

## 1 Introduction

Although essential tremor (ET) is one of the most common hyperkinetic movement disorders (Louis et al., 1998), the underlying disease mechanism is poorly understood. ET has long been considered a benign disorder, but opinions about the disabling nature of ET are changing (Louis and Okun, 2011).

Cerebellar abnormalities are commonly found in investigations of the pathophysiology of ET. Yet, post-mortem studies have provided conflicting results, with cerebellar degeneration reported in some (Louis et al., 2007, Shill et al., 2008) but not all studies (Rajput et al., 2012).

Similarly, neuroimaging results in ET are also incongruent, but do provide support for cerebellar involvement. In structural imaging the most frequent result is cerebellar abnormality in ET, although this is not consistently reported. These reported abnormalities are located in the anterior and posterior lobules of the cerebellum, although not all studies specify the specific areas in the cerebellum (Sharifi et al., 2014). Positron emission tomography experiments consistently report changes in blood flow in the bilateral cerebellum and in some cases in the red nucleus, thalamus and inferior olive during performance of a motor task (Colebatch et al., 1990, Jenkins et al., 1993, Wills et al., 1994). Functional Magnetic Resonance Imaging (fMRI) studies, examining motor tasks in ET patients, report abnormalities in widespread areas of in the (bilateral) cerebellum but are not able to point to specific locations due to methodological limitations (Bucher et al., 1997, Neely et al., 2014). Clinically, cerebellar abnormalities would fit well with the presence of symptoms associated with cerebellar malfunction in ET, such as intention tremor (Deuschl et al., 2000, Louis et al., 2009).

Although many of the results point towards the cerebellum, overall studies and especially functional neuroimaging studies, are inconclusive about the specific areas of the cerebellum involved in ET. One cause contributing to this imprecision in findings may be that ‘ET’ used to be the label for ‘tremor not otherwise specified’, resulting in a heterogeneous group with high variability in clinical presentation, response to therapeutic intervention and on etiologic level. In this study, we have attempted to define a more homogeneous group of ET patients by requiring a clear diagnosis and in addition a positive response to propranolol medication. We selected propranolol for this study, as it is a drug that has obtained level A evidence for efficacy in ET (Zesiewicz et al., 2005).

Moreover, we improved upon existing functional imaging methodology in ET by combining electromyography (EMG) and fMRI. This novel approach allows recording tremor simultaneously with brain activity and to directly relate tremor to brain activity in the analysis. We expected to improve upon the localization of the bilateral cerebellar abnormalities found in earlier fMRI studies.

## 2 Methods

### 2.1 Subjects

This study was conducted in two academic hospitals in The Netherlands: the University Medical Center Groningen (UMCG) and the Academic Medical Center in Amsterdam (AMC). Patients who had a definite diagnosis of ET according to criteria defined by the Tremor Investigation Group were included (Bain et al., 2000). All patients had bilateral upper limb tremor, an age at onset < 65 years, and a disease duration > 5 years. A positive family history was present in most patients (see Table 1) but not required for inclusion. Patients had to report a positive subjective response to propranolol and could not use other tremor medication. Patients and healthy controls (age- and sex-matched) were all right-handed as assessed by the Annett Handedness scale (Annett, 1970) and had a score > 25 on the Mini Mental State Examination to ensure proper understanding of the task. Exclusion criteria were neurological comorbidity (for patients: other than ET), age < 18 years and the use of medication (other than for ET) affecting the central nervous system. The study was approved by the medical ethical committees of the UMCG and the AMC. This study was conducted according to the declaration of Helsinki (Seoul, 2008) and all participants gave written informed consent.

### 2.2 Study set-up

Patients quit propranolol for a minimum of three days before participating in the study; 3-day withdrawal is sufficient to largely eliminate propranolol taking into account the elimination half-life of 3–6 h or 12 h for the long-acting formulation (Hedera et al., 2013). Tremor was assessed off medication using the Fahn–Tolosa–Marin Tremor Rating Scale (TRS) (Fahn, 1993) and a visual analog scale (VAS). The TRS is composed of three parts. Part A consists of assessment of tremor amplitude during rest, posture, movement and finger-to-nose maneuvers. Part B consists of tremor-inducing tasks, including writing, two standardized Archimedes spirals, a line-drawing task and a water pouring task. In part C the patients rate the limitations they experience in daily life due to tremor. Part A and B were performed and videotaped for both hands, separately. An experienced movement disorders specialist (J.D.S.) blindly determined TRS scores for part A and B. The range of the total TRS (part A, B and C) is 0–88. The VAS subjectively rated tremor severity, patients marked a 10 cm line ranging from 0 to 10, 0 meaning no tremor at all and 10 meaning intolerable tremor. In all patients, propranolol was washed in again at the end of the study, according to a personalized schedule under supervision of neurologists specialized in movement disorders (M.A.J.T, K.L.L and A.F.R) in both centers.

### 2.3 Motor tasks during EMG-fMRI

An fMRI scan was performed, while EMG was recorded simultaneously, off-medication. During scanning patients executed a motor task in which they were instructed to alternate periods of 30 s rest with periods of 30 s right hand and arm extension without supporting the hand and arm. The left arm and hand were relaxed which was verified by EMG. An additional task, in which the arm extension was combined with silent reading, was included in the same experiment. To maintain transparency of the results presented here, these blocks were not used for analysis in this study, but were analyzed for a different purpose (Buijink et al., 2015b). Here, we analyzed 10 motor task blocks of 30 s that did not involve reading. Healthy controls mimicked a tremor during right arm extension by self-paced wrist flexion extension. Before scanning, participants were instructed and practiced the task outside the scanner. We asked the participants to raise their arm only slightly, because this was enough to evoke tremor when supine in the scanner. As large movements were also not possible due to limited space in the scanner bore, the disruption of the magnetic field due to movement was limited. All subjects received visual task instruction using slides during scanning.

### 2.4 EMG-fMRI acquisition

Images were acquired on a Philips 3T MR scanner (UMCG: Intera, AMC: Intera and Achieva, Philips, Best, The Netherlands) with SENSE-32 channel (UMCG) and SENSE-16 channel (AMC) head coils. In both centers, T2*-weighted, 3D functional images were obtained using multislice echo planar imaging (EPI) with an echo time (TE) of 30 ms and a repetition time (TR) of 2000 ms. Per TR, 39 axial slices, with a field of view (FOV) of 224 mm, flip angle of 70° with a 64 × 64 matrix and isotropic voxel size of 3.5 × 3.5 × 3.5 mm were acquired. To provide anatomical information, additional T1-weighted 3D anatomical scans with an axial orientation and a matrix size of 256 × 256 mm were obtained (isotropic voxel size 1 × 1 × 1 mm). EMG was recorded simultaneously (BrainProducts GmbH, Munich, Germany (UMCG) and MicroMed, Italy (AMC)), with pairs of sintered silver/silver-chloride MR-compatible EMG electrodes were placed above five right arm muscles: extensor carpi ulnaris (ECU), flexor carpi radialis (FCR), extensor carpi radialis longus (ECRL), flexor carpi ulnaris (FCU) and first dorsal interosseus (FDI). To verify that the left arm and hand were relaxed during the tasks, EMG was recorded from bipolar EMG channels with electrodes placed above three left arm muscles, as well: extensor carpi ulnaris, flexor carpi radialis and first dorsal interosseus. A ground electrode was placed on the left wrist joint. Further EMG recording procedures were similar to the methodology developed in our previous studies (van Rootselaar et al., 2007, van Rootselaar et al., 2008).

### 2.5 EMG-fMRI analysis

**Fig. 1:**
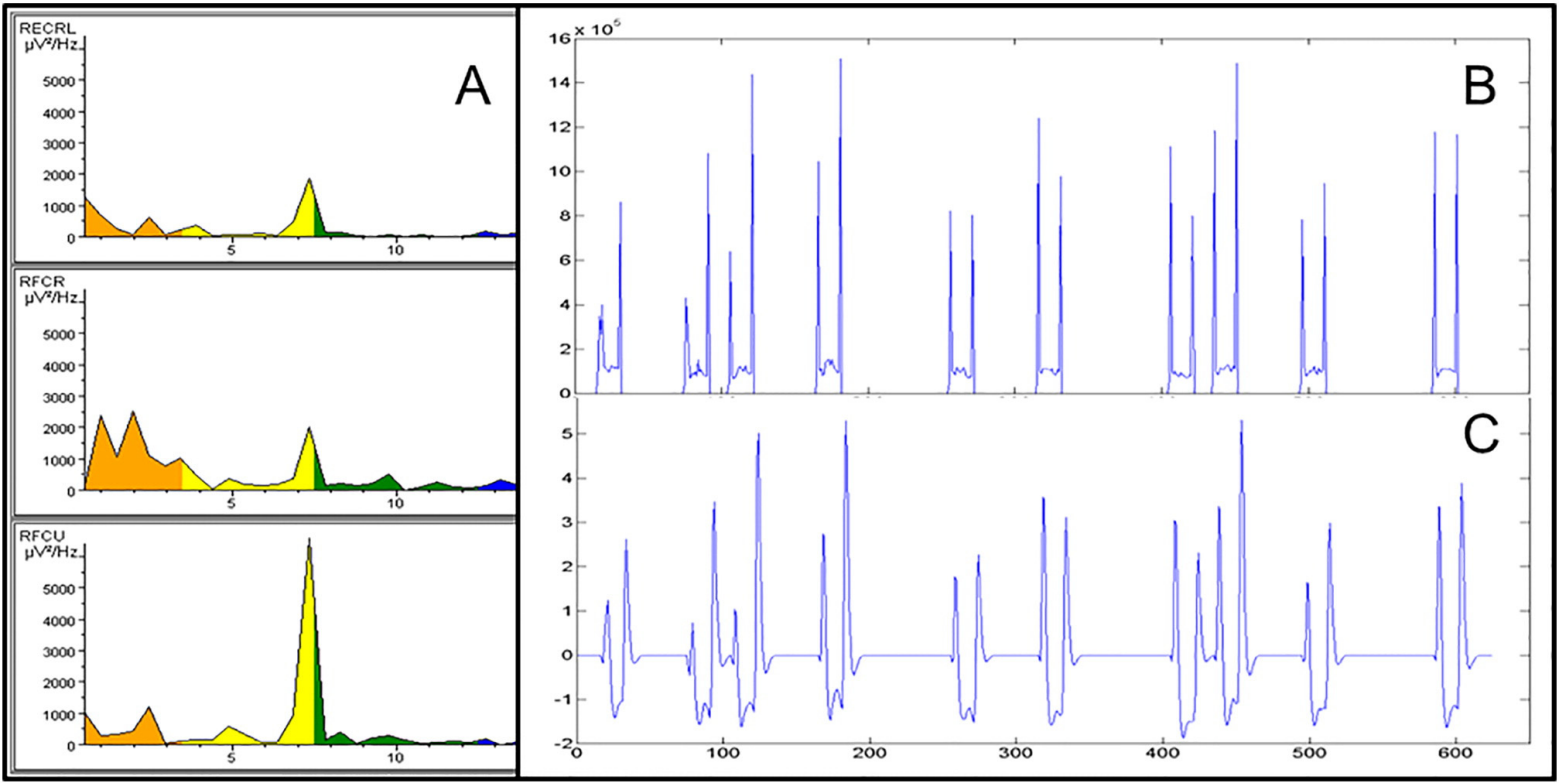
Representative EMG characteristics for one patient. A: Fast Fourier Transform during task of 3 right arm muscles (from top to bottom: ECRL, FCR, FCU). B: EMG regressor: mean EMG per scan of the 3 right arm muscles in A across the task. Higher values are associated with hand and arm extension. C: rEMG regressor as used in the fMRI model: EMG regressor in B after orthogonalization, convolving with the hrf and scaling by its SD.

EMG data were corrected for scanning artifacts using the MR correction algorithms incorporated in Brain Vision Analyzer (Imaging Artifact Reduction method (Allen et al., 2000); UMCG data) and FARM (fMRI artifact reduction for motion (van der Meer et al., 2010); AMC data). After correction, data was further analyzed in Matlab (Matlab R2007a, Mathworks, Natrick, USA) using custom-made scripts. For each segment of 2 s, corresponding to one scan, the frequency spectrum was calculated using the default fast Fourier transform in Matlab (FFT). The individual frequency at the dominant tremor peak (both for tremor and mimicked tremor) was determined for each patient and healthy control by visual inspection of the segments. Patients without a clear and regular tremor peak in the EMG during the task segments were excluded from further analysis. Due to scanner artifacts in the EMG that could not be removed sufficiently by EMG artifact correction, determination of a dominant tremor peak was not possible for these patients. Total spectral power in a 5 Hz symmetrical band around the individual (mimicked) tremor peak frequency was exported for each segment and each right arm EMG channel, resulting in five vectors of the length of the number of scans/segments. The vectors of the three EMG channels with the highest total power were averaged. This procedure resulted in an EMG power vector with one entry for every scan. Next, this vector was orthogonalized with respect to the motor task block vector using Gram-Schmidt orthogonalization, to subtract the information that is already present in the block vector (van Rootselaar et al., 2007). The orthogonalized EMG vector (referred to as residual EMG or r-EMG vector) now provides a measure of additional EMG relative to the mean EMG value across the task block. It represents the variation in tremor intensity over time. Subsequently, the r-EMG vector was element-wise multiplied with the block vector to obtain a vector that only has nonzeroes for the r-EMG during the task blocks, and zeroes otherwise. Finally, this vector was convolved with the canonical HRF, scaled by its SD and used as a regressor in the fMRI design matrix in addition to the block regressor. See Fig. 1 for a representation of EMG characteristics for one patient.

fMRI data were analyzed using SPM8 (Wellcome Trust Centre for Neuroimaging, UCL, London, UK; http://www.fil.ion.ucl.ac.uk/spm). Preprocessing consisted of standard realignment and coregistration steps. A group-specific anatomical template was created using DARTEL (diffeomorphic anatomical registration through exponentiated lie algebra) for a more precise inter-subject alignment to take age-related changes in anatomy into account (Ashburner, 2007). Individual functional data were normalized and smoothed using the DARTEL template and an 8-mm full-width half maximum (FWHM) Gaussian kernel. To reduce movement artifacts, the six movement parameters derived from realignment corrections were entered as covariates in each individual analysis. Inspection of the EMG was used to correct the block regressor for actual on- and offsets of the motor task. Each single-subject first-level model thus consisted of a (corrected) block regressor, a residual-EMG (r-EMG) regressor and the six movement regressors. We refer to the resulting activations as block-related activations and tremor related activations.

In this manner, activations that are common across task blocks will be mostly explained by the block regressor, whereas activations due to variations in tremor intensity will be mostly explained by the rEMG regressor, thereby overcoming the problem in traditional designs used for investigating tremor, where block- and tremor-related activations are mixed.

At second level, dummy variables were inserted in the model, coding for each scanner, to model the effect of the different scanners used in this study. Second level within-group comparisons for the task and r-EMG contrasts, and between-group comparisons for each individual contrast were made on whole brain level. Activations were considered significant at a threshold of p < 0.05 (FWE corrected) and an extent threshold (k) of 20 voxels. This whole brain analysis was only applied for the cerebral hemispheres while cerebellar activations were analyzed with a more specific method for infratentorial regions (see paragraph below).

Given the tremor, it is plausible that the ET patients made more head movements than healthy controls when executing the motor task. To test this we used the scan-by-scan realignment parameters calculated during fMRI preprocessing. We calculated the total range of head motion for each translation direction (x, y and z) and each rotation direction (pitch, roll and yaw) separately, across each session per participant and compared groups.

As we hypothesized cerebellar involvement in ET, we additionally performed an analysis focused on the cerebellum using the Spatially Unbiased Infratentorial Template (SUIT) toolbox (Diedrichsen, 2006). This toolbox isolates the cerebellum and creates a mask. The individual T1 image of the cerebellum was normalized to the SUIT template using nonlinear deformations. The contrast images resulting from the first-level whole-brain analysis were masked with the created cerebellum mask, normalized into SUIT atlas space and smoothed with a Gaussian filter of 4-mm FWHM. Contrasts were thresholded at voxel level p < 0.001, uncorrected, applying a cluster size of 20 voxels.

## 3 Results

### 3.1 Subject characteristics and behavioral results

A total of 40 ET patients were initially included in this study. Data of twenty-one ET patients and twenty-one age- and sex-matched healthy controls were analyzed. Reasons for exclusion of patients from further analysis were either too much head-movement during scanning (one patient), unidentifiable tremor peak during fMRI data collection (16 patients), failure of equipment during scanning (one patient) or incorrect normalization to the DARTEL template (one patient). Analyzed ET patients (12 males) had a mean age of 51.6 (SD 17.8) years and mean disease duration of 29.8 (SD 15) years. Healthy controls (14 males) had a mean age of 50.6 (SD 16.4) years. Age and sex did indeed not differ between the analyzed groups (p = 0.86 and p = 0.35, respectively). Patients had a mean TRS score of 25.7 (SD 10.8) and a mean VAS score of 6.2 (SD 2.1) off medication. Mean peak frequency during scanning was 7.5 Hz in patients and 5.1 Hz in healthy controls. See Table 1 for characteristics of patients and healthy controls. No left arm movement was seen in the EMG signal. Head movement during scanning did not differ between ET patients and healthy controls (see Table 2).

### 3.2 Block-related activations, cerebrum

There was no significant effect of scanner on brain activation, i.e. the results were the same for the different scanners used in this study.

#### 3.2.1 Within-group results

For ET patients, block-related activations (block regressor) were found in the left motor- and premotor cortex and in the supplementary motor area (SMA). Additional activations were found in the right supramarginal gyrus, frontal areas, primary somatosensory cortex, superior parietal cortex and right thalamus (T > 6.49, p < 0.05 FWE, k = 20, see Table 3). In healthy controls, we found block-related activations (block regressor) in the left motor cortex and bilateral premotor cortex and the SMA. In addition, activations were observed in the left supramarginal gyrus, the inferior parietal cortex and frontal regions (T > 6.95, p < 0.05 FWE k = 20, see Table 3).

#### 3.2.2 Between group comparisons

No significant increased activations were detected in ET patients when compared with healthy controls. Healthy controls had increased activations in the left sensori-motor cortex compared to ET patients (both T > 5.34, p < 0.05 FWE, k = 20, see Table 3).

### 3.3 Tremor-related activations, cerebrum

#### 3.3.1 Within-group results

For ET patients, tremor-related activations (r-EMG regressor) were detected in the left motor-, premotor and somatosensory cortex. Additional activations were found in the bilateral visual cortex, the middle part of the cingulate gyrus and the right motor cortex (T > 6.74, p < 0.05 FWE, k = 20, see Table 4). In healthy controls, no significant activations were seen in relation with mimicked tremor (T > 7.05, p < 0.05 FWE, k = 20).

#### 3.3.2 Between-group comparisons

Compared to healthy controls, ET patients showed increased activations in the right motor cortex, middle part of the cingulate gyrus and the left somatosensory cortex (T > 5.45, p < 0.05 FWE, k = 20 see Table 4). The reverse contrast (healthy controls > ET patients) was not further investigated because we found no significant mimicked tremor-related activations in healthy controls.

### 3.4 Block-related activations, cerebellum (SUIT analysis)

**Fig. 2:**
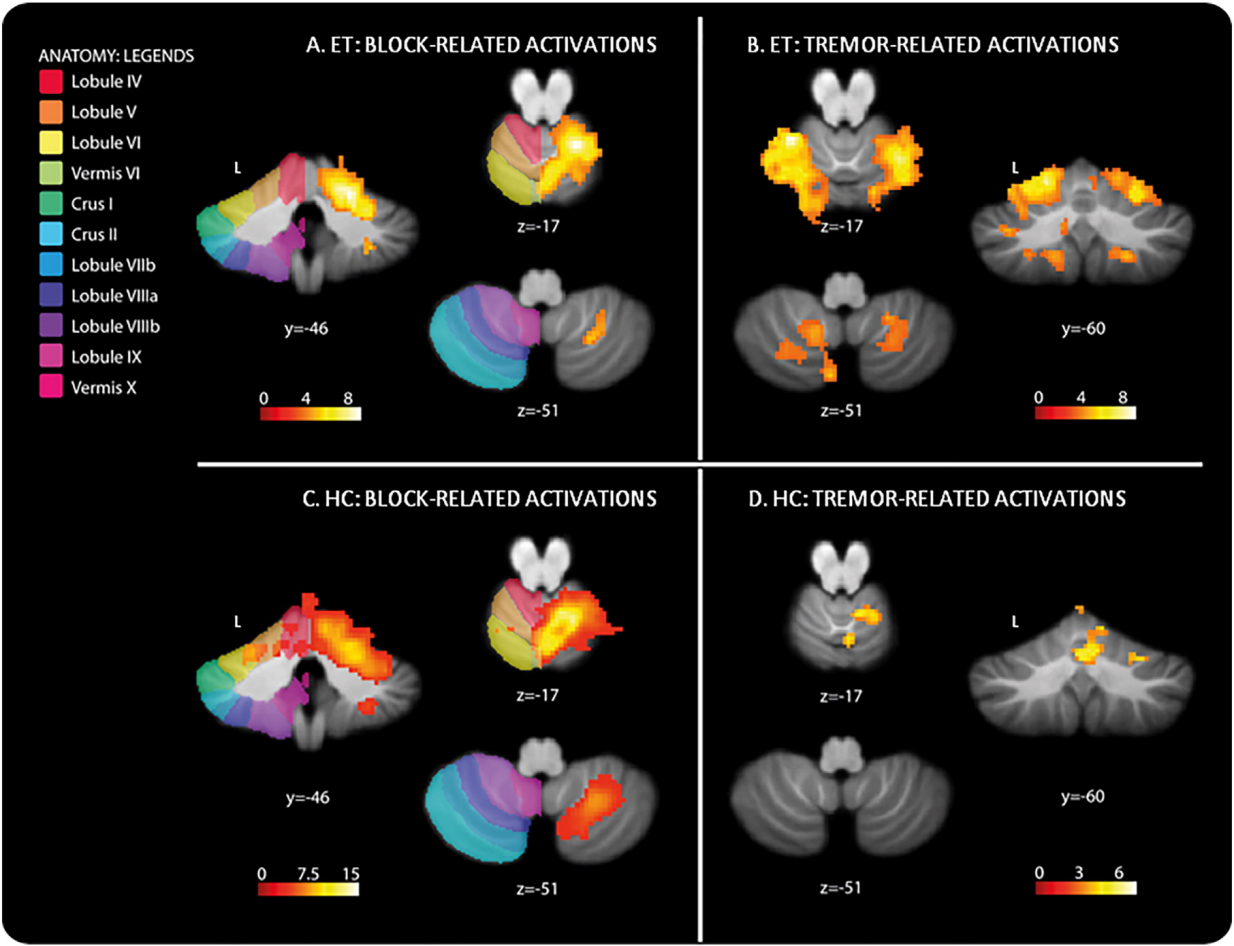
Increased cerebellar activations in essential tremor patients related to the within group comparisons for the block contrast, p < 0.001 (uncorrected, extent k = 20) (1A: block-related activations), and activations related to the within group comparisons for the r-EMG contrast, p < 0.001 (uncorrected, extent k = 20) (1B: tremor-related activations) and increased cerebellar activations in healthy controls related to the within group comparisons for the block contrast, p < 0.001 (uncorrected, extent k = 20) (2A: block-related activations), and activations related to the within group comparisons for the r-EMG contrast, p < 0.001 (uncorrected, extent k = 20) (2B: tremor-related activations). Results are projected on the SUIT-template (Diedrichsen, 2006). The color coded bars at the bottom of the figure indicate SPM T-map intensities. The z-coordinates indicate the position of the transversal planes relative to the anterior commissure–posterior commisure plane. L: left hemisphere. (For interpretation of the references to color in this figure legend, the reader is referred to the web version of this article.)

Block-related activations in the cerebellum of ET patients were found in the right lobule V, VI and VIIIa (T > 3.58, p < 0.001 uncorrected, see Table 5 and Fig. 2A). Healthy controls showed a large cluster of block-related activations in the right lobules V, VI, VIIIa and b (T > 3.61, p < 0.001 uncorrected, see Table 5 and Fig. 2C). ET patients had no increased activations compared to healthy controls in the cerebellum. Healthy controls showed increased activations in the right lobules V, and VI and in Vermis VI compared to ET patients (both T > 3.32, p < 0.001 uncorrected, see Table 5).

### 3.5 Tremor-related activations, cerebellum (SUIT analysis)

Tremor-related activations in the cerebellum were found in left lobules V, VI, VIIb and IX, right lobules V, VI, VIIIa and b and in the brainstem (particularly the dorsomedial parts of the pons and midbrain) in ET patients (T > 3.58, p < 0.001 uncorrected, see Table 5 and Fig. 2B). Healthy controls showed mimicked tremor-related activations in right cerebellar lobule V (T > 3.61, p < 0.001 uncorrected, see Table 5 and Fig. 2D). Increased activations were detected in ET patients when compared with healthy controls in the midbrain, pons and cerebellar lobules V and VIIIb, all left-sided. Healthy controls showed increased activation compared to ET patients in the right crus II (T = 3.32, p < 0.001 uncorrected, see Table 5).

## 4 Discussion

Using a combination of EMG and fMRI we identified specific, somatotopically explicable areas in the bilateral somatomotor regions of the cerebellum associated with tremor. The technique employed here has been used successfully before in a small sample of ET patients, in patients with cortical myoclonic tremor and in Parkinson's tremor (Contarino et al., 2012, Helmich et al., 2011, van Rootselaar et al., 2008). By selecting patients with a clear diagnosis of ET, we aimed to identify brain areas correlating specifically with ET. To our knowledge this is the first controlled EMG-fMRI study investigating a large, more homogeneous group of ET patients.

### 4.1 Block-related brain activations

The participants performed a unilateral (right) arm extension task and, not to our surprise, the classical motor network was activated in both patients and healthy controls. These motor network activations were stronger in healthy participants compared to patients, probably because the tremor simulating movement made by the healthy controls was deliberate and had an observed larger amplitude than the trembling in ET patients. The simulated tremor movements made by the healthy controls were also more constant than the involuntary tremor of the ET patients. The block-regressor therefore likely explained more of the (simulated) tremor-related activation – the part that is common across task blocks — in healthy controls than in ET patients, also probably leading to increased activations.

### 4.2 Tremor-related brain activations

In ET patients, tremor-related activations were found bilaterally in the cerebellum: in left and right lobules VI and V, and additionally in right lobules VIIIa and b, and in the brainstem: in dorsomedial parts of the midbrain, bilaterally, and pons, left-dominant. These results expand on earlier findings that the bilateral cerebellum is involved in ET (Bucher et al., 1997, Buijink et al., 2015a, Neely et al., 2014). Indeed, with our EMG-fMRI approach, we discovered specific, well-defined areas within the cerebellum, thus adding detailed information to the more diffuse localizations that have previously been described.

We identified two distinct tremor related activations in lobules V-VI and in lobule VIII of the right cerebellum, ipsilateral to the right hand and thus particularly implicated in left-hemisphere functions. This particular cerebellar location indeed accurately fits with a previous study on functional connectivity of the cerebral motor hand region which revealed somatomotor regions of the cerebellum (Buckner et al., 2011). In this previous study by Buckner et al. (2011) representation in the cerebellum was cross-lateralized and had a double representation, with a strong primary somatomotor representation in lobules V and VI, and a slightly weaker secondary representation in lobule VIIIb. However, in addition to the cerebellar activity ipsilateral to the tremulous hand, activations were also observed in the contralateral cerebellar hemisphere at the same locations as in the ipsilateral cerebellar hemisphere. Activations were evident in the *left* somatomotor areas, lobules V and VI, and at a lower threshold we found activations in the *left* lobule VIII as well. Thus, we found increased activations in specific somatomotor areas of the *bilateral* cerebellum. The additionally observed increased brainstem activations in the dorsomedial parts of midbrain and pons may point at involvement of the reticular input nuclei of the cerebellum. This putative role of cerebellar input sources in the tremor-related functional changes generates new questions concerning possible underlying causes of pathology to be addressed in future studies. We like to point out, in this respect, that these activations were specifically *tremor*—rather than *movement* related, as the brain activation in these areas covaried with tremor intensity over time *independently* of movement task performance.

In healthy controls activations covarying with simulated tremor intensity were found in the ipsilateral cerebellum, right lobule V. This corresponds to earlier findings in an EMG-fMRI study examining similar motor tasks in healthy participants (van Rootselaar et al., 2007). So, in comparison with simulated tremor, ET patients show additional contralateral activations in the cerebellum.

There are different theories about cerebellar abnormalities in ET. According to the oscillating network hypothesis, tremor is driven by several components in a tremor network, that all act as oscillators (Raethjen and Deuschl, 2012). Within this cerebello-thalamocortical tremor circuit, the cerebellum may act as one of these oscillators. The GABA hypothesis states that ET is associated with GABAergic dysfunction within the cerebellum, as evidenced by increased 11C-flunazenil binding to GABA-receptors in the cerebellar cortex, increasing with tremor severity (Gironell et al., 2012, Gironell, 2014). This leads to a third, neurodegenerative hypothesis, that is based on signs of neurodegeneration such as Purkinje cell loss and torpedoes that have been reported particularly in the cerebellar cortex in ET (Louis et al., 2007, Shill et al., 2008), at the same locations as where GABAergic dysfunction is found.

Our findings could fit in several ways in these hypotheses. We found hyperactivity in multiple parts of the bilateral cerebellum, which could be explained by either primary or secondary effects of ET. Hyperactivity may be primary as a result of neurodegeneration leading to cerebellar atrophy as found in a recent study (Gallea et al., 2015). We hypothesize that the affected cerebellar cells are deficient and disorganized, making them less efficient, and this inefficiency could induce increased activations. On the other hand, the cerebellar hyperactivity may be secondary because of compensation of another defect in the cerebello-thalamocortical tremor circuit.

Bilateral activation in unilaterally challenged tremor may seem odd at first sight. Activation of the right cerebellum is congruent with the right hand and arm extension task and the activated motor cortex in the left hemisphere. Left cerebellar activation points at functional coherence with cortical regions of the right hemisphere, i.e., opposite to the executive motor cortex for right arm movement. In this respect, it is noteworthy that, at a lower threshold, we indeed found increased activations in the right cerebral cortex in ET patients compared to healthy participants. These activations were located in the anterior parietal and premotor cortex. Together, these areas are known to play a major role in sensorimotor transformations underlying task-related visuomotor control (Grafton et al., 1996) and the organization of stereotypic movement (de Jong et al., 2002). Increased coupling between *left* cerebellum and *right* parietal cortex was recently demonstrated by functional imaging investigating multisensory processing (Hagura et al., 2009), independently of right or left arm involvement. One might therefore speculate that ET patients encounter more difficulties in maintaining a steady raised-arm position, which is imaginable because of their tremor, and that the increased activations in the functionally coherent areas of left cerebellar and right anterior parietal and premotor cortex reflect increased higher-order somatosensory processing implicated in motor tuning during posture maintenance.

Whether the activations we found are the cause and/or consequence of tremor cannot be distinguished on the basis of fMRI – which is an associative technique - alone. To make such a distinction, longitudinal studies or interfering techniques such as repetitive transcranial magnetic stimulation might be helpful, but even then, the highly plastic and flexible human brain may make it impossible to make this distinction. Yet, by using a method that focuses on the anatomy of the cerebellum, we were able to more precisely localize the parts in the bilateral cerebellum that are involved in ET.

### 4.3 Methodological considerations

In this study, the use of propranolol was one of the inclusion criteria we applied to define a homogeneous group of ET patients. This is one of the many variables that can be chosen for patient selection. The advantage of choosing this variable is the future option to compare the current propranolol group with other ET patient groups using different medication. This is a first step at an attempt, as far as we know, to differentiate medication-based subtypes of ET.

A common difficulty in fMRI research lies in selecting a suitable task for healthy controls that corresponds well with the patients' task. In this study, a mimicked tremor was used. Consequently, the two groups were actually performing a different task: we asked the tremor patients to maintain their right arm in a postured position, while the healthy controls had to deliberately move their hand. These tasks were chosen to allow optimal distinction of brain networks involved in involuntary tremor as opposed to compensation or afferent feedback by deliberate, mimicked tremor movements. The mimicked tremor movement overall had a slightly lower peak frequency and had a larger flexion–extension movement of the right wrist compared to the tremor in ET patients. The effect of this behavioral difference can be seen in the task-related activations: the healthy controls showed a more widespread and a higher activation signal in comparison with the ET patients. We would like to emphasize however, that for our design and analysis that focuses on identifying tremor-related activations, it is not important that the participants, patient or control, execute the task exactly in the same way, or that their (mimicked) tremor (amplitude or frequency) is the same. Our analysis takes variation in task execution, leading to tremor intensity variation, into account and actually even depends on it. If there would be absolutely no variation in tremor intensity, our r-EMG regressor would be zero and we would not find brain activations related to tremor intensity variability.

Finally, one may wonder why we did not identify activations in the cerebellar nuclei, also hypothesized to be involved in ET generation (Deuschl et al., 2001). Finding activations in small structures, such as the cerebellar nuclei, is a challenge. Spatial resolution and intersubject variation of its shape with functional specialization of subcompartments may play a role in the absence of activation of this cerebellar region with 3-T data acquisition. In addition, high iron content in the deep cerebellar nuclei decreases the blood oxygenation level dependent-signal making functional imaging a difficult matter, that requires not only higher MR field strength but also novel dentate normalization methods (Kuper et al., 2012).

## 5 Conclusions

In the current study, we used EMG-fMRI to identify brain activations specifically associated with variations in tremor intensity in essential tremor patients. Including a more homogeneous patient group and adopting the EMG-fMRI technique for data collection and analysis probably allowed to now identify specific bilateral areas in the cerebellum involved in essential tremor: lobules V, VI and VIII.

## Figures and Tables

**Table 1:** Patients and healthy controls characteristics.

	Age	Sex	Mean tremor frequency (Hz)	Age at onset (years)	Duration (years)	Family history	Head tremor	Alcohol response	Propranolol use (mg)	TRS-score off medication	VAS-score off medication
Patients											
1	21	Male	10	10	11	+	−	+	40	15	5.4
2	22	Male	7	12	10	−	−	+	20	13	5.2
3	27	Male	7.5	0	27	−	−	+	160	22	8.7
4	30	Female	8	15	15	+	−	?	20	17	2.9
5	32	Female	7	3	29	+	−	+	40	22	6
6	35	Male	8	7	28	+	−	?	80	17	7.8
7	46	Male	7.5	5	41	+	−	+	80	19	4.4
8	47	Male	7	15	32	+	−	+	40	15	6
9	48	Female	7	10	38	+	+	−	120	37	5.4
10	53	Female	7.5	28	25	+	+	+	30	35	7.8
11	53	Male	8	16	37	+	−	+	50	19	8.6
12	57	Female	7	22	40	+	+	?	10	23	4
13	62	Female	8.5	5	57	+	−	?	100	36	8.5
14	63	Male	7	43	20	+	−	+	40	17	3.4
15	63	Female	7.5	39	24	+	−	+	80	29	7.4
16	64	Male	6.5	12	52	+	−	+	20	17	4
17	65	Female	7.5	60	5	+	+	?	80	20	2.7
18	69	Male	7.5	40	29	+	+	−	40	45	9.2
19	72	Male	6	10	62	+	+	+	320	48	9.2
20	74	Male	9	50	24	−	−	?	80	32	6.6
21	80	Female	6	60	20	+	−	+	80	41	6.9
Mean (SD)	51.6 (17.8)	M: 12 F: 9	7.5 (0.9)	22 (18.9)	29.8 (15)				72.9 (67.8)	25.7 (10.8)	6.2 (2.1)

HC											
1	20	Male	5								
2	22	Male	3.5								
3	27	Male	5								
4	30	Female	5								
5	33	Female	3.5								
6	36	Male	7.5								
7	47	Male	6								
8	49	Male	4								
9	52	Male	6.5								
10	52	Male	4								
11	56	Male	3.5								
12	57	Female	6								
13	59	Female	5								
14	59	Female	4								
15	60	Male	5.5								
16	60	Female	4								
17	62	Male	4.5								
18	68	Male	5.5								
19	68	Male	5.5								
20	72	Female	7								
21	74	Male	6								
Mean (SD)	50.6 (16.4)	M: 14 F: 7	5.1 (1.2)								

VAS: Visual Analog Scale, range 0–10. TRS: Tremor Rating Scale, range 0–88. SD: standard deviation.

**Table 2:** Statistics for head motion.

	ET mean (SD)	HC mean (SD)	t-Test
Translation x (mm)	0.82 (0.54)	0.85 (0.43)	t(40) = 0.001, p = 0.87
Translation y (mm)	1.09 (0.45)	1.09 (0.76)	t(40) = 0.27, p = 0.97
Translation z (mm)	2.19 (1.30)	2.20 (0.84)	t(40) = 1.26, p = 0.99
Rotation pitch (degrees)	0.05 (0.03)	0.04 (0.03)	t(40) = 1.11, p = 0.60
Rotation roll (degrees)	0.02 (0.01)	0.02 (0.02)	t(40) = 0.08, p = 0.96
Rotation yaw (degrees)	0.02 (0.01)	0.02 (0.01)	t(40) = 0.16, p = 0.61

ET: essential tremor; HC: healthy controls; SD: standard deviation.

**Table 3:** Results for block-related activations (block regressor), cerebrum.

Contrast	Voxels, k	Area	Side	T-value	x^a^	y^a^	z^a^
ET patients^b^	48	Parietal sup	L	7.54	− 26	− 44	66
28	Primary somatosensory cortex	R	7.30	20	− 30	60
3156	Premotor cortex	L	11.21	− 30	− 14	54
sc	SMA	M	10.76	− 6	− 12	54
sc	Frontal sup	R	10.71	18	0	60
636	Supramarginal gyrus	R	9.14	56	− 36	40
sc	Supramarginal gyrus	R	9.12	54	− 24	32
sc	Supramarginal gyrus	R	7.33	48	− 38	44
340	Frontal mid	R	9.12	38	26	34
sc	Frontal mid	R	7.80	32	46	22
sc	Frontal mid	R	7.40	34	42	30
493	Frontal inf, oper	R	10.28	56	12	24
59	Thalamus	R	7.86	20	− 14	20
Healthy controls^b^	4290	Premotor cortex	L	15.97	− 24	− 12	56
sc	SMA	L	15.16	− 4	− 10	56
sc	Medial cingulate gyrus	L	14.20	− 4	− 4	48
45	Parietal inf	L	8.44	− 52	− 22	40
51	Medial cingulate gyrus	R	10.18	16	− 28	38
1546	Supramarginal gyrus	R	11.15	52	− 28	34
sc	Primary somatosensory cortex	R	11.01	36	− 34	50
sc	Supramarginal gyrus	R	10.53	56	− 22	24
162	Rolandic oper	R	9.04	56	4	18
sc	Frontal inf, oper	R	8.98	54	10	26
sc	Premotor cortex	R	8.80	56	4	34
111	Supramarginal gyrus	L	8.27	− 66	− 26	18
sc	Supramarginal gyrus	L	7.98	− 52	− 26	20
sc	Supramarginal gyrus	L	7.64	− 64	− 28	28
ET patients > healthy controls^b^		No significant results					
Healthy controls > ET patients^b^	37	Primary somatosensory cortex	L	7.33	− 38	− 30	56

ET: essential tremor; sc: same cluster; R: right; L: left; M: midline.

a MNI.

b Initial voxel-height threshold p < 0.05 (FWE corrected, extend threshold k = 20 voxels). Coordinates refer to the voxels of maximum activation within clusters of significant activation (p < 0.05, FWE whole brain cluster-level corrected).

**Table 4:** Results for tremor-related activations (r-EMG regressor), cerebrum.

Contrast	Voxels, k	Area	Side	T-value	x^a^	y^a^	z^a^
ET patients^b^	669	Premotor cortex	L	8.38	− 30	− 20	68
sc	Supramarginal gyrus	L	8.28	− 52	− 22	36
sc	Premotor cortex	L	8.13	− 26	− 26	56
104	Precuneus	R	7.63	6	− 40	58
sc	Primary motor cortex	R	7.34	14	− 40	50
sc	Primary motor cortex	R	7.25	12	− 32	54
119	Medial cingulate gyrus	L	8.71	− 12	− 34	46
100	Medial cingulate gyrus	L	7.72	− 2	− 4	42
sc	Medial cingulate gyrus	L	7.48	− 10	− 8	40
106	Primary somatosensory cortex	L	9.81	− 64	− 20	16
sc	Supramarginal gyrus	L	6.85	− 52	− 24	18
103	Medial temporal gyrus	L	8.38	− 30	− 28	12
794	Primary visual cortex	R	8.80	10	− 62	6
sc	Primary visual cortex	R	8.58	10	− 78	6
sc	Primary visual cortex	L	7.99	− 6	− 70	6
74	Associative visual cortex	L	9.16	− 40	− 86	0
23	Associative visual cortex	R	7.77	30	− 84	− 16
Healthy controls^b^		No significant results					
ET patients > Healthy controls^b^	92	Primary motor cortex	R	6.47	10	− 32	52
123	Medial cingulate gyrus	L	6.35	− 10	− 40	52
24	Primary somatosensory cortex	L	6.00	− 48	− 24	52
Healthy controls > ET patients^b^		No significant results					

ET: essential tremor; sc: same cluster; R: right; L: left.

a MNI.

b Initial voxel-height threshold p < 0.05 (FWE corrected, extend threshold k = 20 voxels). Coordinates refer to the voxels of maximum activation within clusters of significant activation (p < 0.05, FWE whole brain cluster-level corrected).

**Table 5:** Results for SUIT-analysis, cerebellum.

Contrast	Voxels, k	Area	Side	T-value	x^a^	y^a^	z^a^
Task-related^b^							
ET patients	1579	Lobule V	R	8.57	20	− 46	− 21
sc	Lobule V	R	7.80	4	− 62	− 23
sc	Lobule VI	R	6.80	28	− 48	− 29
22	Lobule IX	L	4.83	− 4	− 50	− 29
62	Lobule VIIIa	R	5.23	28	− 48	− 47
sc	Lobule VIIIa	R	4.80	22	− 60	− 51
Healthy controls	5187	Vermis VI	R	17.69	6	− 66	− 27
sc	Lobule V	R	13.45	16	− 54	− 17
31	Lobule VIIIa	L	5.47	− 28	− 40	− 43
22	Lobule VIIIa	L	5.60	− 30	− 54	− 53
43	Lobule VIIIb	L	4.62	− 12	− 48	− 57
ET patients > Healthy controls		No significant results					
Healthy controls > ET patients	448	Lobule V	R	6.74	18	− 52	− 25
sc	Vermis VI	R	5.38	4	− 66	− 21
sc	Lobule V	R	4.44	10	− 54	− 13

Tremor related^b^							
ET patients	1903	Lobule V	L	8.48	− 26	− 46	− 15
sc	Lobule VI	L	7.67	− 20	− 62	− 11
sc	Pons	L	6.99	− 8	− 36	− 35
1071	Lobule VI	R	7.23	26	− 54	− 17
sc	Lobule VI	R	6.41	12	− 68	− 9
sc	Lobule VI	R	6.28	28	− 48	− 23
602	Crus II	R	5.30	4	− 76	− 37
sc	Lobule IX	L	5.27	− 16	− 50	− 47
sc	Lobule VIIb	L	4.92	− 6	− 76	− 51
113	Lobule VIIIa	R	4.77	22	− 60	− 49
sc	Lobule VIIIb	R	4,52	20	− 50	− 51
76	Midbrain	R	5.45	8	− 16	3
sc	Midbrain	R	4.99	8	− 26	− 7
sc	Midbrain	R	3.78	4	− 34	1
303	Midbrain	L	5.21	− 10	− 28	− 5
sc	Midbrain	L	4.82	− 20	− 28	− 5
sc	Midbrain	L	4.52	− 12	− 24	3
Healthy controls	75	Lobule V	R	6.29	2	− 74	− 7
sc	Lobule V	R	4.81	0	− 62	− 1
415	Lobule V	R	8.32	4	− 62	− 21
sc	Lobule V	R	5.27	16	− 54	− 21
sc	Lobule V	R	5.17	12	− 56	− 11
ET patients > healthy controls	22	Lobule V	L	3.73	− 16	− 54	− 9
44	Lobule VIIIb	L	4.16	− 16	− 52	− 47
65	Midbrain	L	4.21	− 8	− 28	− 11
sc	Midbrain	L	3.72	− 6	− 18	− 5
42	Pons	L	4.22	− 8	− 34	− 27
Healthy controls > ET patients	10	Crus II	R	3.81	42	− 76	− 47

ET: essential tremor; sc: same cluster; R: right; L: left.

a MNI.

b p < 0.001 (uncorrected, extend threshold k = 10 voxels).
